# Comparative Assessment of Ibuprofen–Arginine and Potassium Nitrate–Fluoride Gels for Reducing Post‐Bleaching Dentin Sensitivity: A Randomized, Double‐Blind, Split‐Mouth Trial

**DOI:** 10.1155/ijod/8196628

**Published:** 2026-06-22

**Authors:** Maria Ritha Veiga Colognese, Larissa Aparecida Kayser, Suelen Kauana de Souza Dalagustinho, Poliana Maria de Faveri Cardoso, Veridiana Camilotti

**Affiliations:** ^1^ Department of Restorative Dentistry, School of Dentistry, Universidade Estadual do Oeste do Paraná (UNIOESTE), Cascavel, Paraná, Brazil; ^2^ Department of Restorative Dentistry, School of Dentistry, Univel University Center, Cascavel, Paraná, Brazil

**Keywords:** arginine, dentin sensitivity, ibuprofen, tooth bleaching

## Abstract

**Objective:**

To compare the effectiveness of ibuprofen with arginine gel and potassium nitrate with sodium fluoride in reducing post‐bleaching dentin hypersensitivity and to assess their effect on tooth color.

**Methodology:**

A randomized, double‐blind, split‐mouth clinical trial was conducted with 30 participants presenting a complete anterior dentition and tooth shade A2 or darker. Each hemi‐arch received one of the desensitizing protocols: 5% ibuprofen + 10% arginine gel (IAG) or 5% potassium nitrate + 2% sodium fluoride (PN). Dentin hypersensitivity was assessed during bleaching and at 1, 24, 48 h, and 7 days post‐treatment using the Visual Analog Scale (VAS). Tooth color was evaluated before and 7 days after bleaching with the Vitapan Classical scale, and the color difference was calculated (ΔSGU and Δ*E*). Data were analyzed using nonparametric tests (paired Wilcoxon and Mann–Whitney), with a significance level of 5%.

**Results:**

Both protocols significantly reduced dentin hypersensitivity during and after bleaching, with no statistically significant differences between groups. Color analysis revealed no intra‐ or intergroup differences, confirming that bleaching efficacy was not affected by either agent. The ibuprofen–arginine gel demonstrated a 79.2% lower cost compared to PN while maintaining equivalent clinical performance.

**Conclusion:**

Ibuprofen–arginine gel and potassium nitrate with sodium fluoride proved equally effective in managing post‐bleaching dentin hypersensitivity without compromising esthetic outcomes. The combination of ibuprofen and arginine represents a safe, cost‐effective, and efficient alternative, offering clinical benefits and expanding therapeutic options for dental practitioners in daily practice.

## 1. Introduction

Dental esthetics is strongly associated with self‐esteem and psychosocial well‐being, exerting a significant impact on individuals’ quality of life [1–3]. In this context, there has been a growing appreciation for dental procedures aimed at enhancing smile harmony. Among these interventions, tooth bleaching stands out as a minimally invasive, safe, and predictable approach capable of providing immediate and long‐lasting esthetic outcomes [4]

Tooth bleaching involves the application of chemical agents capable of penetrating the enamel and reaching the dentin, promoting the oxidation and degradation of chromophore pigments [5]. This procedure can be performed either at home, under the supervision of a dentist, or in the dental office. However, despite its efficacy and predictability, tooth bleaching is associated with relevant adverse effects, with dentin hypersensitivity being the most prevalent complication [6, 7].

Studies report that between 67% and 100% of patients undergoing in‐office bleaching experience some degree of sensitivity, with greater intensity during the first hours after treatment [8]. Although transient, this clinical condition may compromise the patient’s experience and reduce adherence to treatment. Therefore, the effective management of dentin hypersensitivity associated with tooth bleaching represents a highly relevant clinical challenge, with direct implications for dental practice.

Several strategies have been proposed to minimize dentin hypersensitivity, including the use of 5% potassium nitrate and 2% sodium fluoride, applied either alone or incorporated into the bleaching gel [9]. These agents are widely used in clinical practice but show inconsistent efficacy and variable results among patients [4]. This limitation reinforces the need for more effective therapeutic alternatives capable of providing immediate and sustained relief from sensitivity.

In this context, nonsteroidal anti‐inflammatory drugs have been suggested as promising options for the management of dentin hypersensitivity [10]. Ibuprofen has well‐established mechanisms of action, inhibiting prostaglandin synthesis through cyclooxygenase blockade and thereby modulating both the inflammatory process and pain perception [8]. Clinical trials indicate that oral administration of ibuprofen in the preoperative period of tooth bleaching may significantly reduce the intensity of dentin hypersensitivity [8]. However, its systemic absorption is relatively slow, reaching peak plasma concentration only between 1 and 2 h after ingestion, which limits its immediate effectiveness in controlling acute post‐procedural pain [10].

The combination of ibuprofen with arginine represents an innovative pharmacological strategy with the potential to enhance clinical benefits. While ibuprofen modulates inflammation through enzymatic inhibition, arginine, as a nitric oxide precursor, promotes vasodilation, increased local blood flow, and cellular recruitment [8]. These effects support tissue repair and reduce the intensity of the pain response, suggesting a possible synergistic effect between the two compounds. Furthermore, the topical application of this combination allows for a faster onset of analgesic action, minimizing the pharmacokinetic limitations observed with oral administration. Therefore, the aim of the present study is to evaluate the effectiveness of this topical formulation as a desensitizing agent, broadening therapeutic possibilities in dental practice.

## 2. Methodology

### 2.1. Study Design

The present study is a randomized, double‐blind, split‐mouth clinical trial conducted at the Dental Clinic of the Undergraduate Dentistry Program at Centro Universitário UNIVEL, located at Avenida Tito Muffato, 2317, Santa Cruz District, 85,806–080, Cascavel, Paraná, Brazil. The study included a recruitment phase, administration of the clinical protocols, and outcome assessments, and was carried out between February and April 2025. The following outcomes were analyzed: (i) sensitivity during the bleaching procedure; (ii) sensitivity after bleaching within a period of up to seven days; and (iii) changes in tooth color saturation before and after bleaching.

### 2.2. Ethical Criteria

This study was conducted in accordance with the Declaration of Helsinki (1964) and followed CONSORT guidelines. The experimental design was registered in the Brazilian Clinical Trials Registry under the number RBR‐8skkj7q (available at: https://ensaiosclinicos.gov.br/rg/RBR-8skkj7q). The study protocol was submitted to and approved by the Human Research Ethics Committee of UNIVEL and received approval number 7.174.371. All patients meeting the eligibility criteria were informed about the procedure, risks, benefits, and study objectives, and formalized their consent by signing the Informed Consent Form.

### 2.3. Eligibility Criteria

Patients of both sexes, aged between 18 and 30 years, were included to minimize potential biases related to pulp vitality level and responsiveness to painful stimuli. All participants had a complete anterior dentition, vital teeth, no previous history of tooth bleaching or restorations, and central incisors with shade A2 or darker, as determined using the Vitapan Classical scale (Vita, Bad Säckingen, Germany). Shade evaluation was performed along the longitudinal axis of each central incisor in both hemi‐arches under controlled ambient lighting, and records were documented to standardize the inclusion criteria.

To reduce confounding factors that could compromise the assessment of post‐bleaching sensitivity, the following patients were excluded: those with missing anterior teeth, carious lesions, gingival recession, previous restorative or prosthetic treatments, history of dental hypersensitivity, tooth discoloration of systemic origin (e.g., tetracycline or fluorosis), continuous use of anti‐inflammatory or analgesic medications, as well as pregnant or breastfeeding women.

### 2.4. Recruitment

Participants were recruited through advertisements on social media and screened by an examiner familiar with the eligibility criteria. Individuals who met the inclusion criteria and did not present clinical signs listed in the exclusion criteria were invited to participate in the study. Each participant received verbal and written information regarding the nature of the study and the procedures to be performed. However, the process was conducted to ensure that participants remained blinded to their allocation in the experimental groups.

### 2.5. Pilot Study

A pilot study was conducted prior to the main clinical trial to standardize all clinical procedures. This preliminary stage aimed to ensure uniform execution of the desensitizing protocol and the in‐office bleaching technique by the two operators responsible for the interventions to elevate methodological consistency. Dentin hypersensitivity was assessed by a designated examiner using the Visual Analog Scale (VAS), while tooth color evaluation was performed independently with the Vitapan Classical shade guide under standardized conditions. The alignment process focused on procedural reproducibility and protocol adherence rather than on formal statistical assessment of inter‐examiner agreement, as each outcome in the definitive trial was measured by a single blinded assessor. All the information obtained during this pilot study was documented in a digital spreadsheet on the Microsoft Excel software, preserving blinding procedures. No pilot participants were included in the final sample, and no pilot data were incorporated into the main statistical analysis.

### 2.6. Sample Size Calculation

The sample size calculation was performed a priori, considering the split‐mouth design with repeated measures and adopting a superiority hypothesis. The primary outcome used to guide the calculation was dentin hypersensitivity, measured by the VAS during the bleaching procedure. The estimation was conducted using the 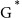
Power software, version 3.1.9.2 (Heinrich‐Heine‐Universität Düsseldorf, Düsseldorf, Germany), based on the *t*‐test family for matched pairs, in order to account for the intra‐individual correlation. An effect size of 0.80 was adopted, based on estimates reported in previous randomized clinical trials evaluating desensitizing protocols for bleaching‐induced sensitivity using similar methodologies and outcome measures [8, 11–14]. Also, a two‐tailed significance level of 5% (α = 0.05) and a statistical power of 80% (1–β = 0.80) were considered. Based on these parameters, the estimated sample size required was 15 participants. However, 30 participants were ultimately included in the study in order to increase statistical precision of the estimates and compensate for potential dropouts.

### 2.7. Randomization and Allocation Concealment

**Figure 1:**
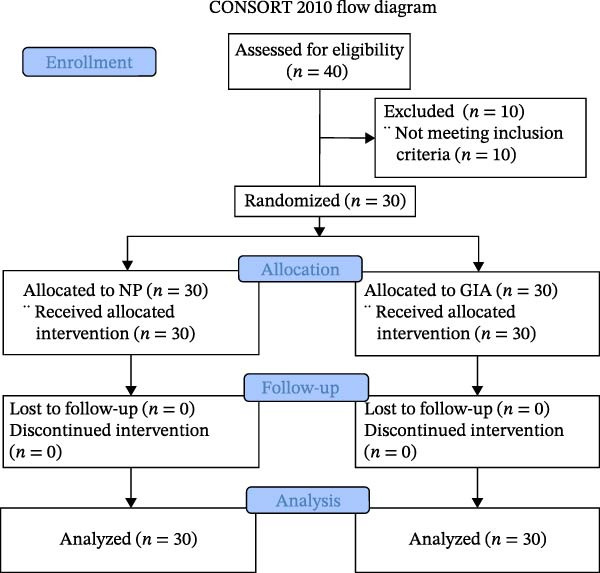
CONSORT flowchart of experimental group allocation and dynamics.

Randomization was performed at the hemi‐arch level within each participant, in accordance with the split‐mouth design, with an allocation ratio of 1:1. For each participant, one hemi‐arch was randomly assigned to the experimental group (ibuprofen 5% + arginine 10% gel—IAG) and the contralateral hemi‐arch was allocated to the control group (potassium nitrate 5% + sodium fluoride 2% gel—PN). allocation sequence was generated prior to the beginning of participant recruitment by an independent researcher who had no involvement in enrollment, clinical procedures, outcome assessment, or statistical analysis. The sequence was created using a computer‐generated random number list using the GraphPad software (GraphPad Software Inc., San Diego, CA, USA), employing simple randomization. The generated list specified, for each sequential participant number, which hemi‐arch would receive the experimental intervention, with the contralateral hemi‐arch automatically assigned to the control intervention. The overall participant flow and allocation process are illustrated in Figure 1.

Allocation concealment was ensured by using sequentially numbered, opaque, light‐impermeable, sealed envelopes prepared by the same independent researcher responsible for sequence generation. Each envelope contained the hemi‐arch assignment corresponding to a single participant and was externally identified only by the participant’s sequential number. The envelopes were stored in a secure location and opened strictly in numerical order, exclusively after confirmation of eligibility criteria, completion of baseline assessments, and signing of the informed consent form. The digital file containing the allocation sequence was protected by password and remained under the exclusive responsibility of the researcher who generated it, and the operator responsible for participant enrollment had no access to this information at any stage of the trial.

### 2.8. Blinding

This study was conducted using a double‐blind design, in which both the participants and the outcome assessors remained unaware of hemi‐arch allocation throughout the trial. The operator responsible for performing the clinical procedures was not blinded to the intervention assignment. However, this operator did not participate in outcome evaluation, data management, or statistical processing. This methodological separation was adopted to control potential sources of bias.

Participants were not informed which hemi‐arch received the experimental intervention or the control intervention, ensuring that pain perception reported during bleaching was not influenced by prior expectations. Dentin hypersensitivity was assessed by a second operator exclusively responsible for applying the VAS, who also remained blinded to hemi‐arch allocation at all time points. Tooth color evaluation was conducted independently using the Vitapan Classical scale under blinded conditions.

The desensitizing gels were prepared, coded, and dispensed into syringes by an independent researcher who had no involvement in participant recruitment, clinical procedures, outcome assessment, or data analysis. Both interventions were placed into identical opaque syringes, standardized in volume, external appearance, labeling format, and application tips, and the materials exhibited similar color and consistency to minimize the possibility of visual differentiation. All data obtained were recorded in a digital spreadsheet using coded group identification to maintain masking during data handling and statistical analysis, and the allocation codes were revealed only after completion of the statistical analysis.

### 2.9. Desensitizing Protocol

All participants initially underwent professional dental prophylaxis using pumice and water for 3 min in each hemi‐arch at a controlled low rotational speed (5.000 rpm). Subsequently, a polyester matrix was placed between the central incisors at the midline and stabilized using a light‐cured gingival barrier, ensuring physical separation of the hemi‐arches during application of the desensitizing agents, ensuring isolation of the treatment fields within the split‐mouth design.

In the control group (PN), a commercially available desensitizing gel containing 5% potassium nitrate and 2% sodium fluoride (Desensibilize KF 0.2%, FGM, Joinville, Brazil) was applied uniformly to the enamel surface using a disposable applicator. The material was maintained in situ for 10 min in accordance with the manufacturer’s instruction and was subsequently removed with an air–water spray for 2 min. In the experimental group (IAG), a compounded gel containing 5% ibuprofen and 10% arginine was employed. The formulation was prepared by a licensed local compounding pharmacy (Vita Fórmula, Toledo, PR, Brazil). The vehicle consisted of propylene glycol as solvent, hydroxyethylcellulose as a viscosity‐enhancing agent, and methylparaben as preservative. The experimental gel was applied using disposable microbrush applicators (Cavibrush, FGM, Joinville, Brazil) with gentle mechanical agitation for 20 s to enhance surface contact and ensure homogeneous distribution of the higher‐viscosity formulation. The material was then maintained on the enamel surface for 10 min, matching the contact time of the control intervention, and removed with an air–water spray for 2 min.

All participants were continuously monitored during the entire procedure for any signs of discomfort, mucosal irritation, or adverse reactions. In the event of moderate or severe pain, the intervention protocol stipulated immediate interruption and appropriate clinical management. However, no adverse events were observed during the study period.

### 2.10. Bleaching Protocol

Following the desensitizing protocol, a light‐cured gingival barrier (Top Dam, FGM, Joinville, Brazil) was applied to protect the gingival tissues. Subsequently, both hemi‐arches were subjected to in‐office bleaching using 35% hydrogen peroxide (Whiteness HP 35% Kit, FGM, Joinville, Brazil) for 45 min, following the manufacturer’s instructions. The bleaching agent was evenly distributed across the buccal surfaces and maintained for the full exposure period without intermediate reapplication. The detailed composition of the materials is presented in Table 1.

**Table 1:** Composition of materials used in the study.

Material	Manufacturer	Composition
Whiteness HP 35% bleaching kit	FGM	35% hydrogen peroxide, thickener, red dye, glycol, and water
Top Dam	FGM	HEMA, urethane dimethacrylate monomer, inert filler, pigments, and photoinitiators
Desensibilize KF 0,2%	FGM	5% potassium nitrate with 2% sodium fluoride
Ibuprofen–arginine gel	Compounded	5% ibuprofen and 10% arginine

### 2.11. Tooth Sensitivity Assessment

**Figure 2:**
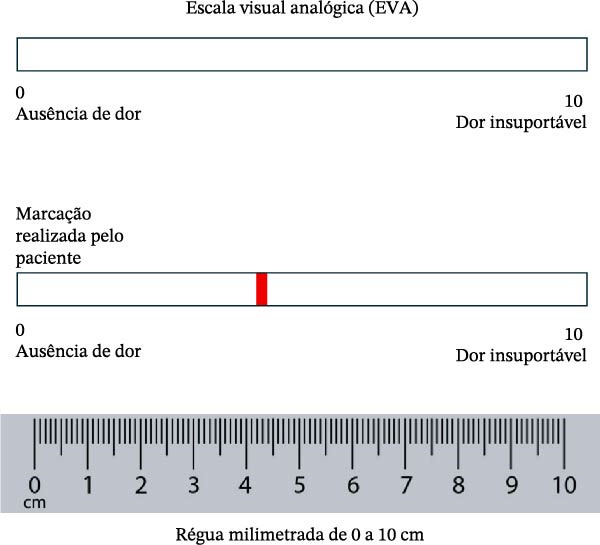
Visual Analog Scale (VAS) for sensitivity assessment.

Dentin hypersensitivity was measured using a 10 cm VAS, illustrated in Figure 2. Participants recorded perceived pain intensity at 5 min intervals throughout the 45 min bleaching session. Additional assessments were conducted at 1, 24, 48 h, and seven days post‐procedure to capture both immediate and delayed sensitivity responses. This repeated‐measures design enabled comprehensive temporal evaluation of the desensitizing effect. To ensure data integrity, received standardized verbal and written instructions regarding post‐procedural care and behavioral restrictions during the evaluation period. They were instructed to refrain from using analgesic, anti‐inflammatory, or desensitizing medications unless strictly necessary. Participants were also instructed to avoid desensitizing toothpastes, fluoride gels, or any other topical agents indicated for dentin hypersensitivity management during follow‐up. Dietary recommendations were consistent with routine post‐bleaching guidance. All participants were explicitly informed of their right to withdraw from the study at any time without penalty or compromise of ongoing dental care, and adverse events were monitored and recorded throughout the follow‐up period.

### 2.12. Tooth Color Assessment

Tooth color was assessed by a previously calibrated examiner using the Vitapan Classical shade guide (Vita, Bad Säckingen, Germany). The maxillary central incisors were used as reference teeth for standardized evaluation. Baseline color assessment was performed prior to bleaching, and a subsequent evaluation was conducted 7 days after treatment completion. All measurements were performed under standardized lighting conditions to minimize environmental variability.

The Vitapan Classical shade guide was selected due to its widespread clinical use and its frequent application in randomized clinical trials investigating tooth bleaching outcomes, which allows comparison with previously published studies. However, it is important to acknowledge that this instrument presents limitations. Because it relies on ordinal categorization rather than continuous color measurement, small chromatic variations may not be fully detected. Furthermore, the chromatic outcome may attain or exceed the highest brightness category of the scale (B1), potentially inducing a ceiling. In addition, the assessment may be influenced by examiner perceptions, lighting variability, differences in viewing distance and angulation, as well as transient alterations in enamel translucency.

Color changes were quantified based on the difference in shade guide units (ΔSGU), using the formula Δ*C* = (Δ*I*)–(Δ*F*). The shade guide was arranged in increasing order of brightness, from the lightest shade (B1) to the darkest (C4), and each shade was assigned to a numerical score. The scores assigned to each shade are detailed in Table 2.

**Table 2:** Scores for shade assessment.

B1	A1	B2	D2	A2	C1	C2	D4	A3	D3	B3	A3,5	B4	C3	A4	C4
1	2	3	4	5	6	7	8	9	10	11	12	13	14	15	16

## 3. Statistical Analysis

### 3.1. Tooth Sensitivity Assessment

The data related to tooth sensitivity were tested for normality and showed a nonparametric distribution. Consequently, nonparametric statistical tests were applied: the paired Wilcoxon test for intragroup comparisons (T1 vs., T2–T14) and the Mann–Whitney test for intergroup comparisons at each time point. The significance level was set at 5% (α = 0.05). Results are presented as median ± interquartile range (IQR). For data interpretation, different lowercase letters indicate statistically significant differences between groups at the same time point, whereas different uppercase letters indicate differences compared with baseline (T1) within the same group.

### 3.2. Tooth Color Assessment

Shade scores were subjected to the Shapiro–Wilk test, which indicated a non‐Gaussian distribution (*p*  < 0.05). Descriptive statistics were presented as median ± IQR. For intragroup comparisons, the Friedman test was used, followed by the paired Wilcoxon test with Bonferroni correction. For intergroup comparisons at each time point, the Mann–Whitney test was applied, also adjusted using the Bonferroni method. The significance level was set at 5% (α = 0.05). All analyses were performed using R software (v. 4.4.0), and the results were validated in IBM SPSS Statistics (v. 29).

## 4. Results

### 4.1. Tooth Sensitivity

Table 3 presents the medians ± IQRs of tooth sensitivity scores for the Potassium Nitrate + Sodium Fluoride group (NP) and the Ibuprofen + Arginine Gel group (GIA) across 14 time points. A significant increase in sensitivity was observed from T2 to T13 compared with baseline (T1), indicated by uppercase letter B, in both groups, with no significant differences between groups (identical lowercase letters). At T14, the scores returned to values comparable to baseline (uppercase letter A).

**Table 3:** Medians ± interquartile range (IQR) of tooth sensitivity scores across 14 time points for NP and GIA groups.

Group	T1	T2	T3	T4	T5	T6	T7	T8	T9	T10	T11	T12	T13	T14
NP	0.00 ± 0.00aA	0.00 ± 0.30aB	0.00 ± 0.30aB	0.00 ± 0.30aB	0.00 ± 0.93aB	0.00 ± 0.80aB	0.00 ± 1.25aB	0.00 ± 0.95aB	0.00 ± 0.60aB	0.25 ± 1.77aB	0.20 ± 2.75aB	0.00 ± 0.15aB	0.00 ± 0.15aB	0.00 ± 0.00aA
GIA	0.0 ± 0.00aA	0.00 ± 0.30aB	0.00 ± 0.30aB	0.00 ± 0.50aB	0.00 ± 0.60aB	0.00 ± 0.70aB	0.00 ± 1.10aB	0.00 ± 0.82aB	0.00 ± 1.08aB	0.30 ± 1.98aB	0.40 ± 1.20aB	0.0 ± 0.15aB	0.0 ± 0.15aB	0.00 ± 0.00aA

*Note:* Different lowercase letters within the same column indicate intergroup differences (Mann–Whitney, *p*  < 0.05). Different uppercase letters within the same row indicate differences compared with Time 1 within the same group (Wilcoxon, *p*  < 0.05).

### 4.2. Tooth Color

The intragroup statistical analysis between Δ*I* and Δ*F* was conducted using the paired Wilcoxon test, revealing statistically significant differences for both the Control Group (NP) and the Test Group (GIA), confirming the effectiveness of tooth whitening in both groups. Intergroup analyses using the Mann–Whitney test showed no statistically significant differences between NP and GIA in any of the three color parameters evaluated. These findings suggest that both desensitizing agents demonstrated equivalent performance regarding whitening efficacy (Tables 4 and 5).

**Table 4:** Median ± interquartile range (IQR) values of shade scores for NP and GIA groups at baseline (Δ*I*) and final (Δ*F*) assessments.

Group	Initial (Δ*I*)	Final (Δ*F*)
NP	5.00 ± 0.00a	2.00 ± 0.00b
GIA	5.00 ± 0.00a	2.00 ± 0.00b

*Note:* Different lowercase letters within the same column indicate intragroup differences (*p*  < 0.05). Different lowercase letters within the same row indicate intergroup differences (*p*  < 0.05).

**Table 5:** Median ± interquartile range (IQR) values of the difference between baseline and final shade (Δ*E*) for NP and GIA groups.

Group	ΔColor (initial–final)
NP	3.00 ± 0.50a
GIA	3.00 ± 0.50a

*Note:* Different lowercase letters within the same row indicate statistically significant differences according to the Wilcoxon test (*p*  < 0.05).

## 5. Discussion

Dentin hypersensitivity remains one of the main adverse effects of tooth whitening, representing a clinically relevant issue that is widely documented in the literature [4]. This discomfort can compromise patient adherence to treatment and reduce satisfaction with esthetic outcomes. In this study, we aimed to evaluate the efficacy of different desensitizing protocols to contribute to the expansion of clinical strategies available for managing this condition.

The results showed that both potassium nitrate combined with sodium fluoride and the ibuprofen–arginine gel were effective in reducing whitening‐induced dentin hypersensitivity, with no statistically significant differences between groups. This finding confirms that both protocols can provide satisfactory outcomes, delivering direct benefits to patients. Furthermore, these observations reinforce that the topical application of desensitizing agents represents an effective strategy compared with systemic medications, which have shown limitations in previous studies [15].

The observed clinical equivalence may be explained by the partial convergence of the mechanisms of action of the two protocols. Potassium nitrate combined with sodium fluoride primarily acts by occluding dentinal tubules, reducing dentin permeability and blocking fluid movement within the tubules [4]. However, although it reduces nerve excitability, this agent does not exert a direct effect on inflammation, a central component of dentin hypersensitivity induced by bleaching agents.

Conversely, ibuprofen combined with arginine acts synergistically in managing this condition. Ibuprofen exerts an anti‐inflammatory effect through the inhibition of prostaglandin synthesis, while arginine stimulates nitric oxide‐mediated vasodilation, enhancing the delivery of immune cells and nutrients and promoting tissue repair. Together, this combination represents a promising strategy for pain relief and reduction of the inflammatory process [8].

Moreover, statistical analysis revealed no significant intra‐ or intergroup differences regarding tooth color change. This evidence confirms that neither agent compromised bleaching efficacy, which is crucial to achieving the desired therapeutic outcome. Thus, both protocols demonstrated satisfactory and consistent performance, providing clinically relevant results for patients.

Additionally, the equivalence between protocols allows clinicians to consider factors such as cost and availability when selecting a desensitizing agent. The ibuprofen–arginine gel was 79.2% less expensive than the control group, which may be decisive in contexts with limited financial resources or within public health programs. This cost advantage, combined with similar clinical efficacy, positions the ibuprofen–arginine gel as a promising alternative for managing dentin hypersensitivity.

In summary, the findings of this study indicate that both desensitizing protocols, potassium nitrate combined with sodium fluoride and ibuprofen–arginine gel, demonstrate comparable efficacy in reducing whitening‐induced dentin hypersensitivity without compromising esthetic outcomes. These results reinforce the clinical relevance of topical desensitizing agents as a safe and effective strategy, and highlight the potential of the ibuprofen–arginine gel as a potentially cost‐effective alternative. This study was appropriately powered, with a sample size determined a priori and further increased to elevate statistical precision, ensuring robust assessment of the primary outcome. Although the follow‐up period was relatively short, it aligns with the study objectives and provides valid data. Future studies with extended follow‐up periods are encouraged to evaluate the durability of the desensitizing effects and their long‐term clinical implications.

## 6. Conclusion

Based on the results of this study, it can be concluded that both ibuprofen–arginine gel and potassium nitrate combined with sodium fluoride are effective interventions for managing dentin hypersensitivity induced by tooth whitening. Both protocols demonstrated the ability to reduce sensitivity while maintaining tooth color stability, reinforcing their safety and clinical acceptability.

## Funding

No funding was received for this manuscript. This study was financed in part by the Coordenação de Aperfeiçoamento de Pessoal de Nível Superior ‐ Brasil (CAPES) ‐ Finance Code 001.

## Disclosure

The authors do not have any financial interest in the companies whose materials are included in this article.

## Consent

All appropriate patient consent forms were obtained.

## Conflicts of Interest

There are no conflicts of interest.

## Data Availability

The data that support the findings of this study are available from the corresponding author upon reasonable request.
